# Regulatory scientists’ work has important ramifications for public health and should be open to public scrutiny

**DOI:** 10.1186/s12961-018-0371-4

**Published:** 2018-11-15

**Authors:** Shai Mulinari, Courtney Davis

**Affiliations:** 1 Department of Sociology, Faculty of Social Sciences, Lund University, Box 117, SE-221 00 Lund, Sweden; 2 Department of Global Health and Social Medicine, Faculty of Social Science and Public Policy, Kings College London, London, United Kingdom

## Abstract

The Swedish Medical Products Agency (MPA) objects to the fact that we occasionally refer to one of its senior ex-employees by name. However, names of individual MPA assessors, Food and Drug Administration (FDA) reviewers, and European Medicines Agency rapporteurs and co-rapporteurs are cited in regulatory documents and are a matter of public record. In our paper (*Health Res Policy Syst* 15:93, 2017), we in no way suggest that regulatory decisions were left to individual reviewers or assessors, although we do emphasise that individual MPA and FDA employees’ scientific assessments and benefit–risk evaluations are critical to the decision-making process. In this response to the MPA, we raise a further issue – one in which the question of personal identification of individuals is relevant – and this pertains to the accountability of influential scientists and experts who contribute to public policy decisions with important ramifications for public health. In our view, it is important that interested observers are able to identify those influential individuals, and entirely appropriate that their work should be open to public scrutiny.

## Letter to the Editor

The names of individual Swedish Medical Products Agency (MPA) assessors, Food and Drug Administration (FDA) reviewers, and European Medicines Agency rapporteurs and co-rapporteurs are cited in regulatory documents and are a matter of public record. We are therefore perplexed by the MPA Director’s complaint that “*By naming a specific assessor at the MPA, the reader is given the false impression that a single employee is solely responsible for the benefit–risk evaluation of a drug*” [1]. Throughout our paper, we alternate between referring to an individual by their name and referring to them by their institutional title – as is common in academic analyses and historical accounts of organisational decision-making. However, we in no way suggest that regulatory decisions are left to individual reviewers or assessors. On the contrary, we clearly state that an MPA Board “*reviewed all MPA expert assessments*” ([2], p. 7) and our paper attributes final regulatory decisions to the FDA, the MPA, or the European Medicines Agency – not to individuals. For example, we write that “*the MPA went on to formally conclude that, overall, the evidence showed that Relenza alleviated symptoms of influenza 1–2.5 days quicker than placebo, which justified marketing authorisation*” ([2], p. 7). And although we emphasise that individual MPA and FDA employees’ scientific assessments and benefit–risk evaluations are critical to the decision-making process, the central argument of our paper is that such evaluations must be understood in the context of broader institutional cultures, practices and resources.

We are further puzzled by the MPA’s objection to the fact that we occasionally refer to Dr Uhnoo by name (rather than exclusively by her institutional title) since the test of whether or not we imply that an individual bears sole responsibility for regulatory approval decisions in Sweden does not depend on whether that individual is identified personally, by name, or generically in terms of their organisational role. Regardless, and for the reasons stated above, the MPA’s accusation that we misrepresent the regulatory process is unfounded. However, we would like to raise a further issue – one in which the question of personal identification of individuals is indeed relevant – and this pertains to the accountability of influential scientists and experts who contribute to public policy decisions with important ramifications for public health. In our view, it is important that interested observers are able to identify those influential individuals, and entirely appropriate that their work should be open to public scrutiny.

Dr Uhnoo is no exception to this rule. As a recognised expert in the field of infectious diseases, clinical virology and vaccinology, she has occupied numerous influential positions, including the position of Programme Manager for the national vaccination program in Sweden 2011–2016 [3]. During the time of the Relenza evaluation, she served as Scientific Secretary to the Swedish Association of Infectious Diseases and, on behalf of this organisation, was a member of the Swedish Reference group for Antiviral Therapy between 1997 and 2011 [3]. Dr Uhnoo has co-authored national treatment guidelines for influenza [4, 5], and in 2005 she authored the scientific report on pandemic stockpiling of antivirals [6], which informed the Swedish Government’s decision in 2005 and 2006 to buy neuraminidase inhibitors to treat 20–25% of the country’s population [7]. In these documents and publications, she identifies herself as a Senior Expert and Clinical Assessor at the MPA and as Associate Professor or Professor and Senior Physician at Uppsala University Hospital. Following the norms of scientific writing and exchange, and also in recognition of Dr Uhnoo’s considerable influence over key public health decisions – both as a prominent scientist and as a senior civil servant – we consider her naming in the text as unproblematic.
